# GMFilter and SXTestPlate: software tools for improving the SNPlex™ genotyping system

**DOI:** 10.1186/1471-2105-10-81

**Published:** 2009-03-09

**Authors:** Markus Teuber, Michael H Wenz, Stefan Schreiber, Andre Franke

**Affiliations:** 1 Institute for Clinical Molecular Biology, Christian-Albrechts-University Kiel, Arnold-Heller-Str. 3, D-24105 Kiel, Germany; 2 Applied Biosystems, 850 Lincoln Center Drive, Foster City, California 94404, USA; 3 Department of General Internal Medicine, University Clinic S-H, Campus Kiel, Arnold-Heller-Str. 3, D-24105 Kiel, Germany

## Abstract

**Background:**

Genotyping of single-nucleotide polymorphisms (SNPs) is a fundamental technology in modern genetics. The SNPlex™ mid-throughput genotyping system (Applied Biosystems, Foster City, CA, USA) enables the multiplexed genotyping of up to 48 SNPs simultaneously in a single DNA sample. The high level of automation and the large amount of data produced in a high-throughput laboratory require advanced software tools for quality control and workflow management.

**Results:**

We have developed two programs, which address two main aspects of quality control in a SNPlex™ genotyping environment: GMFilter improves the analysis of SNPlex™ plates by removing wells with a low overall signal intensity. It enables scientists to automatically process the raw data in a standardized way before analyzing a plate with the proprietary GeneMapper software from Applied Biosystems. SXTestPlate examines the genotype concordance of a SNPlex™ test plate, which was typed with a control SNP set. This program allows for regular quality control checks of a SNPlex™ genotyping platform. It is compatible to other genotyping methods as well.

**Conclusion:**

GMFilter and SXTestPlate provide a valuable tool set for laboratories engaged in genotyping based on the SNPlex™ system. The programs enhance the analysis of SNPlex™ plates with the GeneMapper software and enable scientists to evaluate the performance of their genotyping platform.

## Background

Single-nucleotide polymorphisms (SNPs) are the most frequent variation in the human genome and are therefore preferably used for mapping in genetic research. SNP genotyping has become an essential tool for investigating the genetic background of complex diseases and for analyzing genetic variation between individuals. Different genotyping systems are available, depending primarily on the scale of throughput. Laboratories equipped with low-throughput genotyping platforms often use the TaqMan^® ^system, while for higher throughput the SNPlex™ system is one of the main tools for SNP genotyping [1,2]. SNPlex™ is a cost-efficient, highly flexible and scalable technique, which is based on oligonucleotide ligation (OLA), multiplex polymerase chain reaction (PCR) and capillary electrophoresis. SNPlex™ assays are combined in a SNP set, which allows for simultaneous genotyping of up to 48 SNPs. Eighty percent of SNPs in non-repetitive regions of the human genome are suitable for SNPlex™ assays [2]. The analysis software GeneMapper generates genotypes from electrophoretic raw data. It supports automated allele calling, visualization and quality control of genotype data.

At the Institute for Clinical Molecular Biology in Kiel we operate a SNPlex™ genotyping platform, which produces up to 280,000 genotypes per day. This huge amount of results requires strategies for ensuring quality control and correctness of data. Here we present two programs, which address issues specific for the SNPlex™ system.

One key feature of the GeneMapper software is the automated calling of alleles. However, the employed clustering algorithm often produces false positive results, if too many wells have a low signal intensity. The analysis can significantly be improved by removing those wells from the raw data. We have developed GMFilter to automate this task.

Applied Biosystems offers a test system for evaluating the genotype accuracy of SNPlex™. This system consists of the SNPlex™ System gDNA Plates Kit and the SNPlex™ System Control Pool Kit. Because no software for analyzing a test plate is available, we have developed SXTestPlate. The program calculates the call rate and concordance for each DNA of the test plate and each SNP of the control pool. SXTestPlate can be used independently of the Applied Biosystems test kits, which allows scientists to use custom plates and control pools.

Both programs are intended to reduce genotyping errors. A genotyping error is defined as the discrepancy between the observed genotype and the true genotype. Such errors can seriously affect linkage analysis, decrease the power of association studies or cause incorrect allele identification in population genetic studies. Pompanon *et al*. provide an in-depth overview of the cause and consequences of genotyping errors and how to quantify error rates [3].

## Implementation

GMFilter and SXTestPlate have been written in Visual Basic 6. They were implemented on a Microsoft Windows 2000 system and run on Windows 2000 and XP platforms. SXTestPlate requires a free Microsoft SQL Server 2005 Express Edition database. It connects to the database via TCP/IP using the OleDb provider. For performance reasons the file import in SXTestPlate uses the bulk insert function of the database. In order to execute bulk insert commands users must belong to the bulkadmin or sysadmin fixed server role. The online help for GMFilter and SXTestPlate is based on Microsoft HTML Help 1.4 and has been written with Microsoft HTML Help Workshop. Both programs are free software, which can be redistributed and/or modified under the terms of the GNU Lesser General Public License.

Our SNPlex™ genotyping platform uses 384-well DNA plates. The detection is done with Applied Biosystems 3730*xl *DNA Analyzers, which are equipped with 96-capillary arrays. The machines are controlled by the Data Collection 3.0 software. Analysis is performed with the GeneMapper 4.0 software.

## Results and discussion

### GMFilter: a tool for improving GeneMapper analysis

Under optimal conditions, when most of the SNPlex™ assays perform well and when the DNA samples are of superior quality, i.e. giving rise to high signal intensities, the clustering algorithm of the GeneMapper software performs adequately. Bad wells are assigned a well quality of zero and are consequently ignored in the analysis. However, if too many wells have low signal intensities, GeneMapper tends to include bad wells in the analysis, which can lead to false positive genotype assignments. We found that removing bad wells (corresponding to bad samples) from the raw data reduces noise, which significantly improves the analysis. For an automated processing of this task we have developed GMFilter.

**Figure 1:**
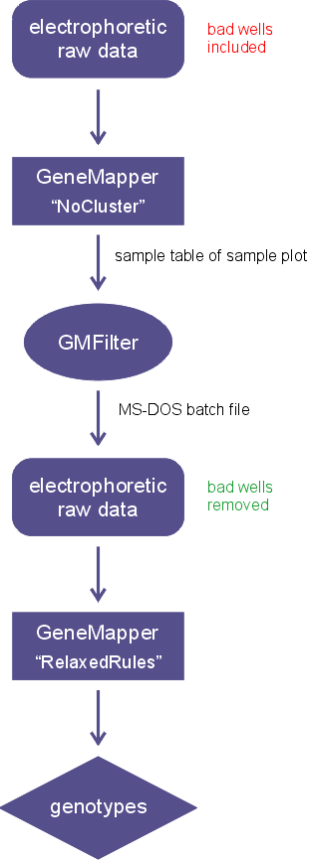
**Workflow of GMFilter usage**. GMFilter removes bad wells from the raw data, which were identified by the "NoCluster" analysis method. A subsequent reanalysis with the "RelaxedRules" method leads to improved clustering results.

**Figure 2:**
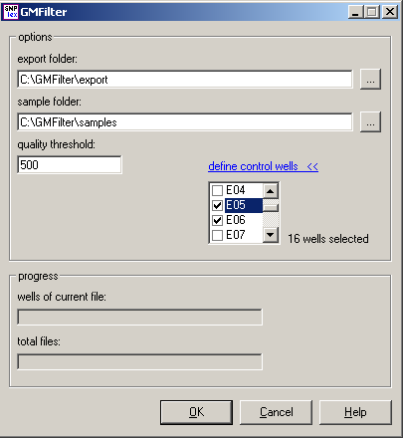
**Screenshot of GMFilter**. Users can specify the threshold for the median signal intensity and which wells are being used as positive and negative controls.

**Figure 3:**
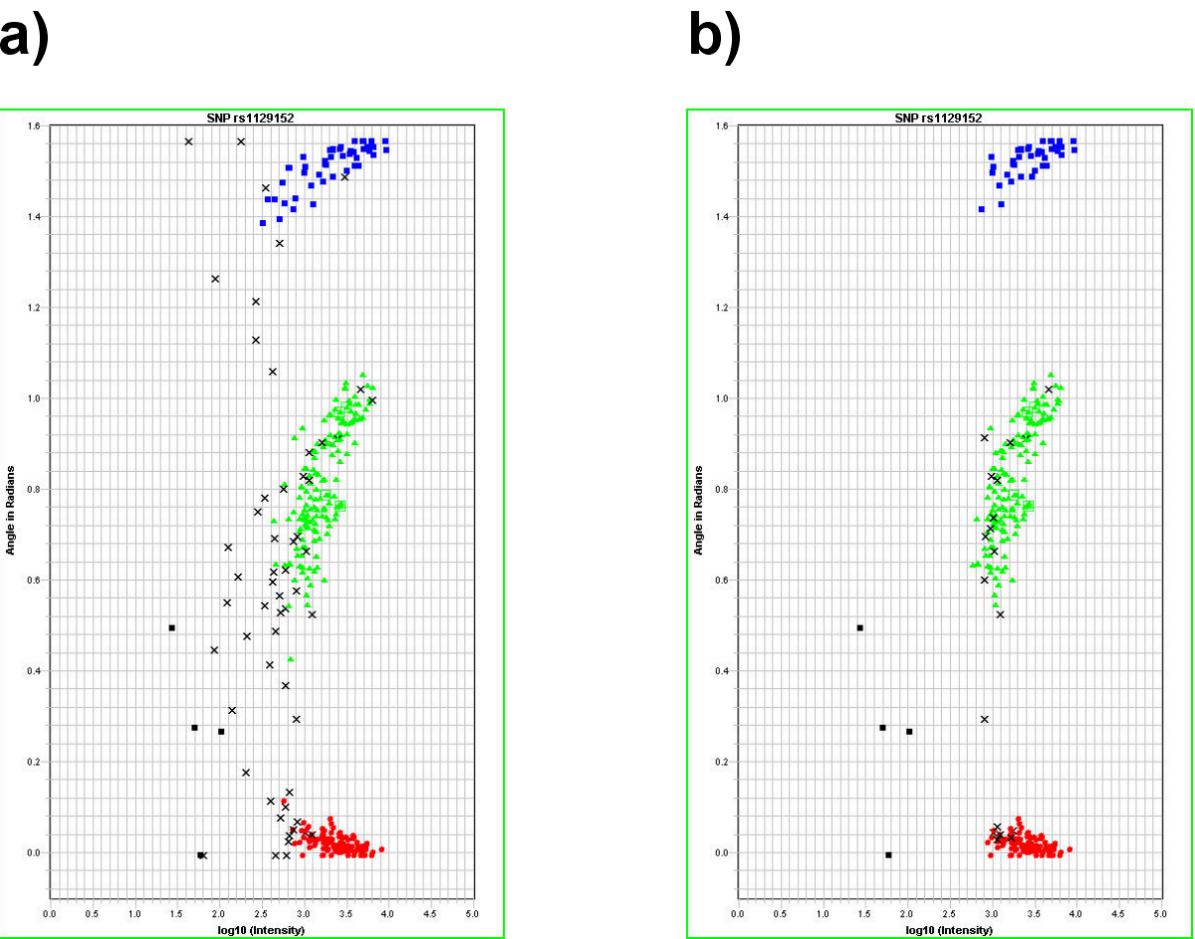
**Cluster plots from the GeneMapper software before and after processing a plate with GMFilter**. Unfiltered data (a) contains considerably more noise than after processing with GMFilter (b). The recommended threshold of 750 has been used. For the cluster plots, all four plate quadrants have been overlaid in GeneMapper. Black squares represent negative controls (no-template controls) and black crosses uncalled genotypes.

Figure 1 shows the workflow of the GMFilter usage: Users first analyze a SNPlex™ plate with a special GeneMapper analysis method ("NoCluster") in order to identify bad wells. In GMFilter they enter the folders, which contain the exported sample plot table from GeneMapper and the raw data (fsa files). The tool lets users specify the threshold for the median signal intensity and which wells are being used for controls (Figure 2). GMFilter analyzes the exported sample plot table by importing the file and converting it to an unbound recordset for better internal data handling. It calculates the median signal intensity for each well using basic descriptive statistics. Based on these calculations it generates a normal MS-DOS batch file, which deletes fsa files of wells having a median signal intensity below the specified quality threshold. Deletion of bad wells and reanalysis of the plate with a modified version of the standard Rules analysis method ("RelaxedRules") in GeneMapper leads to much better clustering results (Figure 3). GMFilter needs about 10 minutes for processing the sample plot table of a 384-well plate on a normal desktop computer (Windows XP, 2 GHz processor, 512 MB RAM). The analysis methods "NoCluster" and "RelaxedRules" are available on the GMFilter homepage [4].

### SXTestPlate: a tool for evaluating genotyping performance

Regular checks of the genotyping platform are an important aspect of quality control. To facilitate this task Applied Biosystems offers the SNPlex™ System gDNA Plates Kit and the SNPlex™ System Control Pool Kit. In combination they are a useful tool for evaluating genotype accuracy and precision, e.g. detection of potential contamination. The control pool contains 48 assays and the test plate contains 44 distinct DNA samples, which are replicated multiple times within one quadrant. Applied Biosystems also provides a file with the "true" reference genotypes. However, evaluating a test run by comparing text files in Excel, for example, can be a tedious work. We thus developed SXTestPlate as a user friendly tool for the statistical analysis of genotypes from the test plate, which was typed with the control pool.

SXTestPlate also supports custom test plates and control pools, making the tool independent of the Applied Biosystems kits. The DNA samples on the test plate may be replicated multiple times. SXTestPlate determines automatically, how often a sample is replicated, by grouping the imported genotypes by identical DNA and SNP identifiers. This flexibility makes the program applicable for genotyping technologies other than SNPlex™ as well.

**Figure 4:**
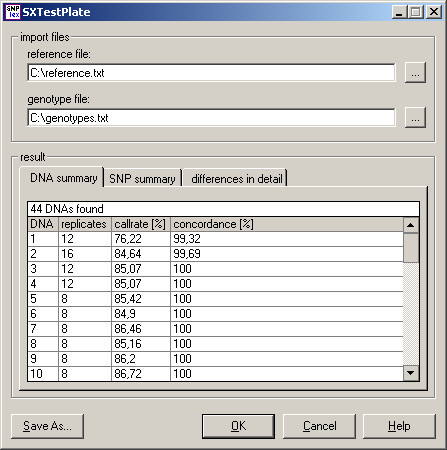
**Screenshot of SXTestPlate**. The tool reports the genotype call rate and concordance for each DNA and SNP. In addition it shows the detailed genotype discrepancies.

SXTestPlate imports the reference genotypes and the exported genotype table from the GeneMapper software into an SQL Server 2005 Express Edition database and compares them to each other by using complex structured query language (SQL) statements, which are executed as stored procedures on the database server. The tool reports the call rate and the concordance with the expected genotypes for each DNA and SNP (Figure 4). If discrepancies are found, detailed results are shown. On a normal desktop computer (Windows XP, 2 GHz processor, 512 MB RAM) SXTestPlate needs approximately 10–15 seconds for analyzing a 384-well plate, which was typed with a control pool containing 48 assays.

## Conclusion

Laboratories engaged in high-throughput SNP genotyping have a great demand for automated data management in order to handle the large amount of genotyping data and to ensure quality control. We have developed GMFilter and SXTestPlate as a useful toolkit for SNPlex™ genotyping platforms. Both programs are freely available for non-commercial use.

We have demonstrated that removing wells with a low signal intensity from the raw data of a SNPlex™ run considerably improves the analysis of the GeneMapper software, since it reduces noise and consequently, the number of false positive genotype assignments. GMFilter has been developed as a user-friendly Windows program for the automated filtering of SNPlex™ raw data.

An important issue in a high-throughput laboratory is the routine evaluation of the genotyping performance. Users can make a test run with the SNPlex™ System gDNA Plates Kit and the SNPlex™ System Control Pool Kit offered by Applied Biosystems or with custom plates and SNP sets. We have developed SXTestPlate for the statistical analysis of a test run. The tool reports the call rate and concordance for each DNA of the test plate and each SNP of the control pool.

## Availability and requirements

Project homepages

GMFilter: , SXTestPlate: 

Operating system

GMFilter: Windows NT or later versions

SXTestPlate: Windows 2000 or later versions

Programming language

Visual Basic 6

Other requirements

SXTestPlate requires the free database SQL Server 2005 Express Edition from Microsoft .

License

GNU Lesser General Public License

## Authors' contributions

MT implemented the software and wrote the manuscript. MW helped to develop the GeneMapper analysis methods for GMFilter. SS supervised the genotyping and helped writing the paper. AF performed the genotyping, helped to design the software and contributed to the writing of the manuscript. All authors read and approved the final manuscript.
